# Acceleration of Vascular Sprouting from Fabricated Perfusable Vascular-Like Structures

**DOI:** 10.1371/journal.pone.0123735

**Published:** 2015-04-10

**Authors:** Tatsuya Osaki, Takahiro Kakegawa, Tatsuto Kageyama, Junko Enomoto, Tadashi Nittami, Junji Fukuda

**Affiliations:** 1 Graduate School of Pure and Applied Sciences, University of Tsukuba, Tsukuba, Japan; 2 Faculty of Engineering, Yokohama National University, Yokohama, Japan; Osaka University, JAPAN

## Abstract

Fabrication of vascular networks is essential for engineering three-dimensional thick tissues and organs in the emerging fields of tissue engineering and regenerative medicine. In this study, we describe the fabrication of perfusable vascular-like structures by transferring endothelial cells using an electrochemical reaction as well as acceleration of subsequent endothelial sprouting by two stimuli: phorbol 12-myristate 13-acetate (PMA) and fluidic shear stress. The electrochemical transfer of cells was achieved using an oligopeptide that formed a dense molecular layer on a gold surface and was then electrochemically desorbed from the surface. Human umbilical vein endothelial cells (HUVECs), adhered to gold-coated needles (ϕ600 μm) via the oligopeptide, were transferred to collagen gel along with electrochemical desorption of the molecular layer, resulting in the formation of endothelial cell-lined vascular-like structures. In the following culture, the endothelial cells migrated into the collagen gel and formed branched luminal structures. However, this branching process was strikingly slow (>14 d) and the cell layers on the internal surfaces became disrupted in some regions. To address these issues, we examined the effects of the protein kinase C (PKC) activator, PMA, and shear stress generated by medium flow. Addition of PMA at an optimum concentration significantly accelerated migration, vascular network formation, and its stabilization. Exposure to shear stress reoriented the cells in the direction of the medium flow and further accelerated vascular network formation. Because of the synergistic effects, HUVECs began to sprout as early as 3 d of perfusion culture and neighboring vascular-like structures were bridged within 5 d. Although further investigations of vascular functions need to be performed, this approach may be an effective strategy for rapid fabrication of perfusable microvascular networks when engineering three-dimensional fully vascularized tissues and organs.

## Introduction

Regenerative medicine has attracted increasing attention as a new therapy that circumvents the shortage of donor organs for transplantation and can potentially cure various severe diseases by using a patient’s own cells or other immunologically matched stem cells [1,2]. Thin and avascular tissues such as skin [3], cartilage [4], and the cornea [5] have been successfully treated using engineered cellular replacements. However, engineering of thick and cell-dense organs such as the liver and pancreas have lagged behind. One major issue is the lack of suitable approaches for the fabrication of vasculatures. Thick organs in the body rely on the supply of oxygen and nutrients from vascular networks. Cells residing more than a few hundred micrometers away from the nearest capillary cannot survive [3,6]. Thus, a reliable strategy for the fabrication of spatially aligned vascular networks *in vitro* is desired in order to engineer three-dimensional (3D) thick tissues and organs for regenerative medicine.

Many attempts at vascular network fabrication have been reported. The typical approach is *in vitro* prevascularization, a method in which endothelial cells, randomly embedded in a hydrogel with parenchymal cells such as hepatocytes, are allowed to spontaneously form vascular networks in culture prior to transplantation [7–10]. However, because this approach depends on endothelial cell self-organization, the networks are inhomogeneous and culture medium cannot be perfused. Therefore, only limited amounts of oxygen and nutrients are supplied by molecular diffusion. Without perfusion, the supply of sufficient oxygen and nutrients to millimeter- and centimeter-thick dense tissues poses a challenge. An alternative approach is to fabricate microchannels in the hydrogel by using sacrificial templates [11–13] or needles and subsequently seed endothelial cells inside these microchannels [14–17]. Using this approach, sufficient oxygen and nutrients can be delivered to parenchymal cells by convective flow of culture medium through the microchannel. However, one possible drawback is that, because medium flow has to be stopped for at least a few hours to allow endothelial cells attach onto the internal surfaces of microchannels, parenchymal cells are subjected to hypoxic cell death and severe ischemia-reperfusion damage at the beginning of the culture period [18,19]. Therefore, a relatively rapid fabrication method for perfusable vasculatures is desirable.

We have reported an approach previously in which perfusable and endothelialized microchannels were fabricated rapidly using electrochemical cell transfer from a template, without the occurrence of significant hypoxic cell damage. This cell transfer was mediated by an electrochemical reaction between gold-coated glass rods and electrically responsive molecules [20–23]. In this approach, electrically responsive molecules were pre-absorbed onto gold-coated glass rods via gold-thiolate bonds, forming a self-assembled monolayer. Endothelial cells adhering to the molecular layer on the rod surfaces were transferred from the rods to the internal surface of microchannels in a collagen gel within 5 min by applying an electrical potential that cleaved the gold-thiolate bond and reductively desorbed the molecular layer. The fabricated perfusable vascular structures were ~600 μm in diameter and aligned at 500-μm interspaces in two dimensions. During the subsequent culture period, endothelial cells migrated into the hydrogel and formed luminal structures. However, this approach is limited to the fabrication of 2D aligned vascular structures, because it is challenging to rapidly align and extract individual rods simultaneously after the application of the potential. Therefore, in this study, by using a multi-needle in which multiple conductive needles were arranged in a spatially controlled manner, we expanded this strategy to engineer 3D aligned vascular-like structures. In addition, to accelerate angiogenic sprouting and self-organization of transferred endothelial cells, we examined the synergistic effects of a culture medium additive and application of shear stress by subjecting the structure to medium flow.

Growth factors including vascular endothelial growth factor (VEGF), fibroblast growth factor (FGF), and platelet-derived growth factor are primarily responsible for regulating sprouting and vascularization [24–27]. VEGF, the best-studied vascular morphogen, initiates sprouting by promoting the formation of tip cells [28,29]. In a previous study, we used culture medium supplemented with VEGF and FGF-2, but sprouting and long-term stabilization were not achieved in a highly reproducible manner [21]. Phorbol 12-myristate 13-acetate (PMA) is known to strongly enhance the angiogenic ability of HUVECs and is a low-molecular weight drug that is currently undergoing clinical trials as a therapy for hematologic cancer and bone marrow disorders (ClinicalTrials.gov Identifier: NCT00004058) [30]. Shear stress is another key factor affecting vascularization. Although some studies have reported that shear stress significantly induces angiogenesis of HUVECs [31], another study has shown that it attenuates angiogenesis [32]. In the present study, we examined the effects of PMA and shear stress on sprouting as well as their synergistic effects in our system with the aim of finding a new approach for engineering vascularized 3D large and thick tissues and organs.

## Material and Methods

### Materials and regents

The materials used for culture substrate fabrication were obtained from the following commercial sources: single-needle (glass; diameter, 600 μm; length, 3.2 cm) from Hirschmann Laborgeräte (Eberstadt, Germany); multi-needle (stainless steel; 9 needles; diameter, 500 μm; pitch, 500 μm; length, 1 cm) from Musashi Engineering, Inc., (Tokyo, Japan); synthetic oligopeptide CGGGKEKEKEKGRGDSP from Sigma-Aldrich (St. Louis, MO, USA); and poly(methyl methacrylate) plates from Comnet (Koube, Japan). The cells and regents used for cell culture were as follows: human umbilical vein endothelial cells (HUVECs, CC-2517A) from Cambrex Bio Science (Walkersville, MD, USA); immortalized HUVECs constitutively expressing green fluorescent protein (GFP-HUVECs), a generous gift from Dr. J. Folkman (Boston Children’s Hospital, Boston, MA, USA); endothelial basal medium (EBM-2, CC-3156) and SingleQuots growth supplement (CC-4176) from Lonza (Basel, Switzerland); type I collagen (Cellmatrix Type I-A) from Nitta Gelatin (Yao, Japan); Ham’s F12 medium and phosphate-buffered saline (PBS) solution from Invitrogen (Waltham, MA, USA); and PMA from Sigma-Aldrich. All other chemicals were purchased from Wako Pure Chemicals Industries (Odawara, Japan), unless otherwise indicated.

### Oligopeptide design and modification of gold surfaces

**Fig 1:**
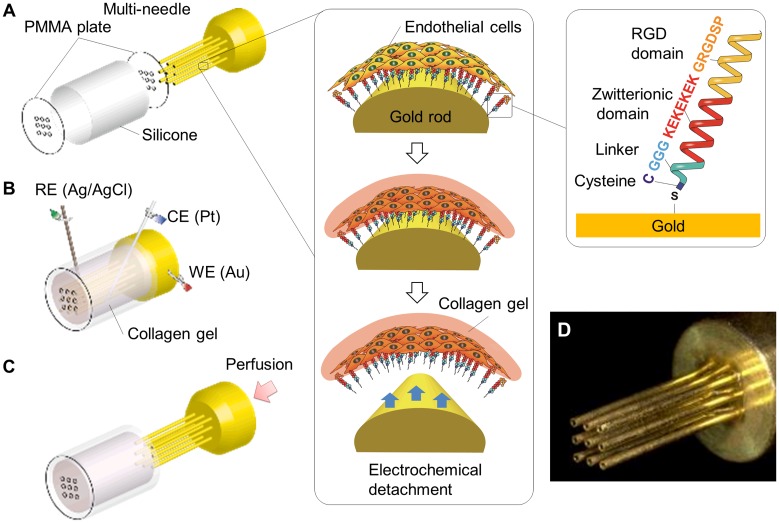
Fabrication of perfusable vascular-like structures by using an electrochemical reaction. (A) Modification of the multi-needle surface with zwitterionic oligopeptides to mediate endothelial cell adhesion. (B) Placement of the HUVEC-coated needle in a culture chamber. Pouring and gelation of collagen in the chamber. Application of electric potential to transfer HUVECs to the internal surface of the microchannels within the collagen gel and desorption of the molecular layer. (C) Needle removal and culture medium perfusion. (D) Gold-coated multi-needle (9 needles; diameter, 500 μm; pitch, 500 μm; length, 1 cm).

The oligopeptide CGGGKEKEKEKGRGDSP was designed to generate a self-assembled monolayer on a gold surface to mediate cell adhesion, while preventing nonspecific protein adsorption (Fig 1). The oligopeptide consists of three functional domains. The cysteine (C) residue has a thiol group that spontaneously forms a gold–thiolate (Au–S) bond on a gold surface. The domain containing alternating glutamic acid (E) and lysine (K) residues was designed to induce close packing of the molecular layer via electrostatic intermolecular interactions [23]. This neutrally charged zwitterionic oligopeptide modification provided a non-fouling surface [33]. The GRGDSP domain promotes integrin-mediated cell adhesion [34].

The gold surfaces were prepared by sputter-coating single needles with Cr (1-nm-thick) and Au (100-nm-thick) and multi-needles with Au (200-nm-thick). Notably, the modification of a gold surface with this oligopeptide does not require any organic chemistry and was performed by simply immersing the gold-coated needles in 0.5 mM oligopeptide aqueous solution at 4°C for 12 h. The modified surface was rinsed with pure water, sterilized with 70% ethanol, and was then washed three times with PBS before cell seeding.

### Cell preparation and seeding on oligopeptide-modified gold needles

GFP-HUVECs and HUVECs were grown in EBM-2 supplemented with SingleQuots growth supplement at 37°C and 5% CO_2_. HUVECs from passages 3 through 7 were used for experiments. Cells were passaged using 0.05% trypsin-EDTA before cells reached 80% confluence. The oligopeptide-modified multi-needle was placed in a T-25 culture flask and HUVECs were seeded at a density of 7.5 × 10^5^ cells/flask. After 1 d of culture, the multi-needle was transferred to another T-25 flask to remove any unattached cells. HUVECs were also seeded on the oligopeptide-modified single needles in a cell-nonadherent 35-mm dish (Sumitomo Bakelite Co., LTD, Japan) and seeding at a density of 2.0 × 10^5^ cells/dish. After 12 h of culture, excess cells were removed by exchanging the culture medium. The culture medium for the multi- and single needles was changed every 48 h until confluence (3–5 d).

### Culture chambers

A cylindrical culture chamber was designed to transfer HUVECs from the multi-needle and to perfuse culture medium (Fig 1A). The chamber consisted of two poly(methyl methacrylate) plates, 9 mm in diameter with 9 holes that were ~600 μm in diameter, and a silicone cylinder with an 8-mm internal diameter and 1-cm-length. The plates and their holes were fabricated from a l-mm-thick flat plate by using a computer-aided laser machine (Laser PRO C180; GCC, Taiwan). Because the diameter of the plates was slightly larger than that of the silicone cylinder, the plates were fixed at both ends of the cylinder by simply inserting the plates to the silicone cylinder. The chamber volume was ~500 μL.

To monitor changes in cell morphology over the course of the culture period, a square culture chamber in which three single needles could be fixed was also designed. The square chamber was fabricated by assembling 6 poly(methyl methacrylate) plates. The volume of the chamber was ~250 μL and its internal height, width, and depth were 8 mm, 10 mm, and 3 mm, respectively. Using the computer-aided laser machine, 3 holes that were 600 μm in diameter at 500-μm-intervals were fabricated on two opposite plates. These holes were used as a guide to spatially align 3 single needles in the chamber and to extract them straight from the chamber.

### Fabrication of vascular-like structures

Vascular-like structures in the cylindrical and square chambers were fabricated in a similar manner except that the cylindrical chamber was used for the multi-needle whereas the square chamber was used for the single needles. Schematic diagrams of the procedures performed using the cylindrical chamber are illustrated in Fig 1. The multi-needle with a confluent HUVEC layer was fixed at the end of the silicone cylinder with one poly(methyl methacrylate) plate. After a collagen solution (2.4 mg/mL, 500 μl) was poured into the cylindrical chamber, the chamber was closed with the other poly(methyl methacrylate) plate (Fig 1B). The chamber was then incubated for 20 min at 37°C. After collagen gelation, Ag/AgCl reference and Pt counter electrode wires were inserted into the chamber through the silicone cylinder. After the application of a potential of -1.0 V for 5 min with respect to the reference electrode, the multi-needle was carefully removed from the chamber (Fig 1B).

For perfusion culture, the microchannels were connected to a micro-syringe pump through the holes fabricated in the poly(methyl methacrylate) plates. Culture medium was perfused at 1 and 10 μL/min through each microchannel. HUVECs on the surfaces of the microchannels in the square chamber were visualized using phase-contrast and fluorescent microscopy (IX-71; Olympus, Japan) as well as using confocal laser microscopy (LSM700, Carl Zeiss, Germany).

### Effects of PMA on sprouting of HUVECs

To assess the effects of PMA exposure on sprouting, HUVECs (1.0 × 10^5^ cells) were seeded in a 35-mm dish coated with collagen gel (2.4 mg/mL, 1 mL) and were exposed to 0, 20, 50, 250, 500, and 1000 ng/mL of PMA in culture medium for 7 d [35]. Cell morphology in the collagen gel was recorded every 2 d by performing phase-contrast microscopy.

### HUVEC orientation under perfusion culture conditions

Under perfusion culture conditions, HUVECs lining the microchannels were exposed to shear stress generated by a medium flow of 1 and 10 μL/min through each microchannel before and 12 h after perfusion. Phase-contrast images were collected, binarized, and converted to a power spectrum by performing two dimensional Fourier transforms. The power spectrum was then converted to a histogram of polar coordinates to obtain orientation intensity, the ratio of short to long axes from the histogram [36,37]. Statistical significance was determined by performing ANOVAs with Tukey–Kramer’s multiple comparisons test (*P < 0.05, **P < 0.01) on data collected from three independent experiments.

### Fluorescence staining

Cells were fixed with 4% paraformaldehyde in PBS for 15 min, treated with 0.2% Triton X100 for 10 min, and then stained with rhodamine phalloidin (Cytoskeleton, Inc., Denver, CO, USA) and DAPI for 20 min at room temperature. After washing cells three times with PBS, cells were examined using a fluorescence microscope (IX-71, Olympus).

### Quantification of capillary length

HUVECs in the collagen gel within square chambers were examined using a confocal laser microscope. Capillary length and sprouting branch number were analyzed using image analysis software (IMARIS, Bitplane, Switzerland) as described elsewhere [38]. For 3D rendering, acquired high-resolution confocal images were stacked and 3D structures were generated using isosurface rendering. The total capillary lengths, branching points, and average segment lengths were quantified from the reconstructed 3D images by using the filament tracer module of the software.

## Results and Discussion

### Fabrication of endothelialized microchannels in collagen gel

**Fig 2:**
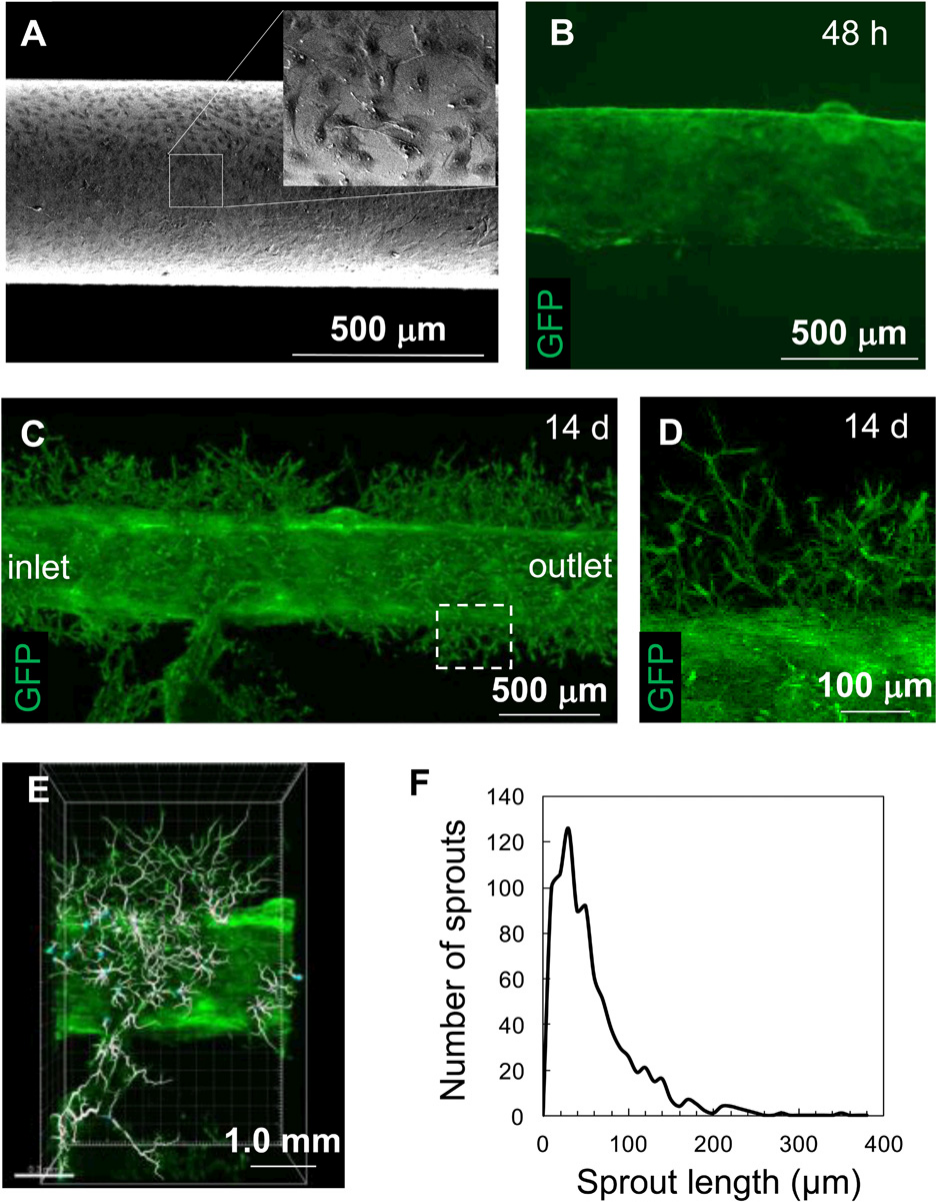
Endothelial cell sprouting in collagen gel under stationary culture conditions in a culture dish. (A) SEM image of HUVECs on a gold rod prior to cell transfer. The inset is a magnified view. (B) HUVECs transferred onto the internal surface of a microchannel in collagen gel after 48 h of culture. (C, D) HUVECs migrating into the collagen gel at 14 d. The box in (C) indicates the location of (D). (E, F) Image analysis. Sprouts were traced and quantified using image analysis software (E, S1 Movie). The sprout length was quantified at 21 d (F).

HUVECs were seeded on gold-coated single needles modified with oligopeptides and were grown until they reached confluence (at least 3 d, Fig 2A). The HUVEC layer was successfully transferred to the inner surface of the microchannels in the collagen gel along with electrochemical desorption of the oligopeptide (Fig 2B). The entire process including collagen gelation and HUVEC transfer was completed within 30 min. The collagen gel was then removed from the chamber and cultured in a conventional culture dish so that sufficient oxygen and nutrients were supplied from the surface of the collagen gel in the absence of culture medium perfusion. The transferred HUVECs spontaneously migrated up to 250 μm away from the microchannel surfaces into the collagen gel at 14 d of culture (Figs 2C and 2D). The extent of sprouting and branching increased over time and the maximum and average capillary lengths were approximately 350 μm and 75.4 μm, respectively, at 21 d of culture (Figs 2E and 2F, see also S1 Movie). This accelerated sprouting process was attributed to the presence of growth factors such as VEGF and basic FGF, and others in the serum used to supplement the EGM-2 culture medium. However, considering that organ-specific cells will be encapsulated and grown in a collagen gel in order to engineer 3D cell-dense tissues and organs, the sprouting process would likely be too slow to supply sufficient oxygen and nutrients to these cells. Because cell survival depends on the supply of oxygen and nutrients, and proliferative cells typically double within a few days, we next sought to accelerate vascular network formation.

### Effect of PMA on sprouting

**Fig 3:**
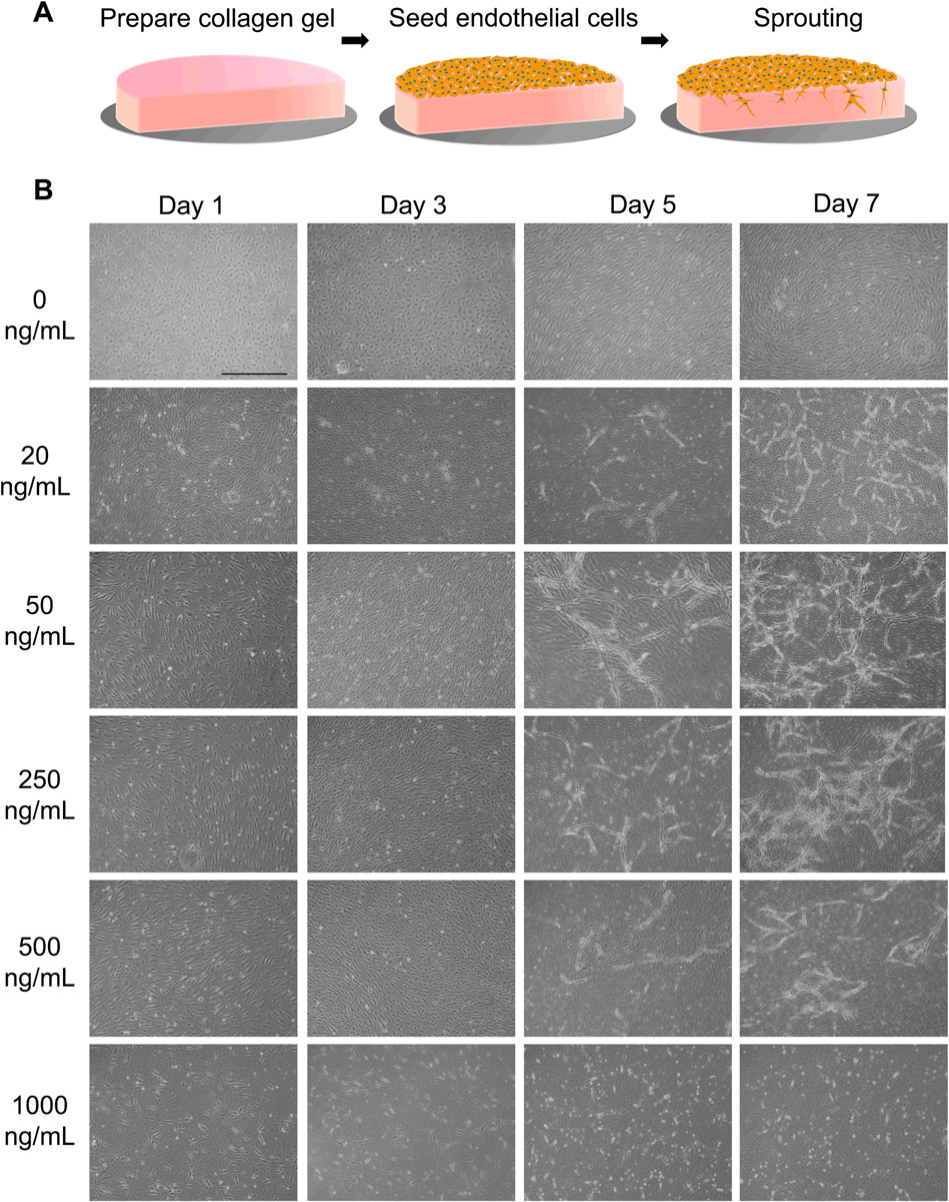
Effects of PMA on endothelial cell vascularization in collagen gel. (A) Schematic diagrams of the experimental design. (B) Phase-contrast microscopy images of HUVECs exposed to various PMA concentrations. HUVECs did not sprout into the collagen gel in the absence of PMA, forming a monolayer on the surface. When exposed to 20–500 ng/mL PMA, HUVECs formed typical luminal structures. At 1000 ng/mL PMA, sprouting was inhibited and cells became detached. Scale bar, 1 mm.

PMA is a strong inducer of sprouting angiogenesis [39]. It disrupts the integrity of the endothelial cell layer and upregulates the synthesis of proteinases, such as matrix metalloproteinases, that are essential for cell invasion in the early stages of angiogenesis [40,41]. To clarify effects of PMA on endothelial cells sprouting, HUVECs seeded on a collagen gel were exposed to various PMA concentrations (Fig 3A). In the absence of PMA, HUVECs grew only on the surface of the collagen gel and reached confluence. They formed random patterns on the surface, but did not migrate into the collagen gel for at least 7 d of culture (Fig 3B). In the presence of 20–500 ng/mL PMA, HUVECs migrated into the collagen gel and formed luminal structures within 7 d of culture. Although no significant difference in luminal structure formation were observed between the cells exposed to 20 and 250 ng/mL PMA, this process was mildly inhibited in the presence of 500 ng/mL PMA. Exposure of cells to 1000 ng/mL PMA completely inhibited luminal structure formation, and some cells became detached from the surface. PMA is also a strong inducer of apoptosis. PMA induces apoptosis by activating caspase-3 via the PKC/nuclear factor-kappa B (NF-κB) signaling pathway, and its effective concentration in endothelial cells is reportedly higher than 100 ng/mL [42,43]. In this study, however, no apparent apoptotic cell death was observed with exposure up to 500 ng/mL. We attribute this to the effect of FGF-2 present in the EGM-2 medium, because FGF-2 inhibits the PKC/NF-κB pathway via downregulation of the Bcl-2 family [44]. Based on these results, we opted to use 50 ng/mL PMA in subsequent experiments.

### Effect of shear stress on cell orientation

**Fig 4:**
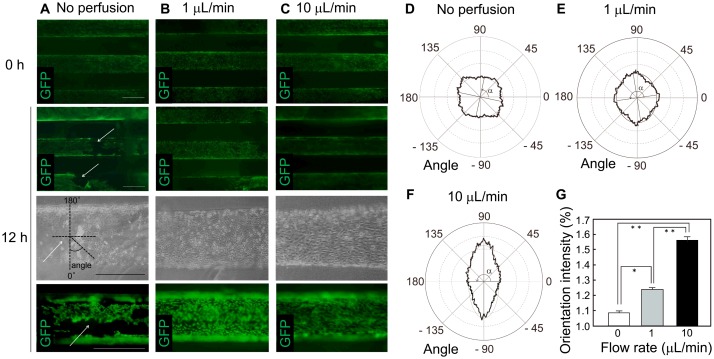
Effects of shear stress on endothelial cell alignment. (A–C) HUVEC layers electrochemically transferred to the internal surfaces of microchannels and cultured in chambers for 12 h in the presence or absence of culture medium perfusion. HUVECs were partially detached in the absence of perfusion (A, arrows), but continued to cover the surface at a flow rate of 1 μL/min (B) and 10 μL/min (C). Scale bars, 500 μm. (D–G) Quantification of HUVEC orientation with respect to flow direction after 12 h of culture in the presence or absence of perfusion. The angle and mean amplitude at 0 μL/min (D), 1 μL/min (E), and 10 μL/min (F), and comparisons of the orientation intensity (G). *P < 0.05, **P < 0.01. Error bars indicate standard deviations calculated from three independent experiments.

After the electrochemical transfer of HUVECs, the collagen gel was kept in the chamber and HUVECs enveloping the internal surfaces of the microchannels were exposed to shear stress generated by culture medium perfusion. In the absence of perfusion, some regions of the HUVEC layers were disrupted after 12 h of culture (Fig 4A). As shown in Fig 2C, HUVECs retained their layers in the microchannels and migrated into the collagen gel when it was removed from the chamber and cultured in medium in the absence of perfusion. These results suggest that oxygen shortage occurred during the 12-h culture period, even at a very low density of HUVECs and no parenchymal cells present in the hydrogel. Under perfusion culture conditions of 1 and 10 μL/min through each microchannel, the HUVEC layers were maintained (Fig 4B and 4C). Perfusion changed the orientation of HUVECs in a flow rate-dependent manner toward the direction of flow within the microchannels (Fig 4D–4G). The most marked reorientation was observed at a flow rate of 10 μL/min, where the shear stress generated corresponded to one-tenth of that observed in the body (in *in vivo* microvascular venous, 1–5 dyn/cm^2^) [45,46]. The endothelial cell orientation under such low shear stress has been controversial. Endothelial cells have been reported to be oriented at 0.1–2.5 dyn/cm^2^ in microfluidic devices [47]. However, another report has demonstrated that at least 1 dyn/cm^2^ was required for cell orientation [48]. Such differences may be attributed to the differences in culture environments, including configuration of microchannels, e.g. circular microchannels, and culture additives, such as PMA and other growth factors. We also tested the effect of a flow rate of 100 μL/min, which generates shear stress equivalent to that in *in vivo* microvascular venous (~1.2 dyn/cm^2^), and observed a complete collapse of the HUVEC layers (data not shown). To engineer more robust vascular structures, additional strategies such as gradual increases in flow rate and fabrication of additional layers, such as smooth muscle cell layers, are warranted.

### Synergistic effect of PMA and shear stress exposure

Angiogenesis has been extensively studied in the field of cancer research and its complex processes are becoming increasingly understood at the molecular level [49]. Sprouting is the initial event in angiogenesis and accompanies endothelial cell migration and proliferation. PMA and shear stress may activate these two endothelial cell processes through different signaling pathways. PMA upregulates PKC. By inhibiting the apoptosis-associated PKC/NF-κB pathway by using FGF-2 as described above, the signal becomes transduced though mitrogen-activeted protein kinase kinase 1 and 2, resulting in growth activation. Shear stress upregulates nitric oxide synthase, which catalyzes nitric oxide, a signaling molecule that not only enhances mitrogen-activeted protein kinase kinase 1 and 2 activity and activates cell proliferation, but also stimulates cell migration [50–52]. Therefore, we expected PMA and shear stress to have synergistic effects on the acceleration of sprouting in our system.

**Fig 5:**
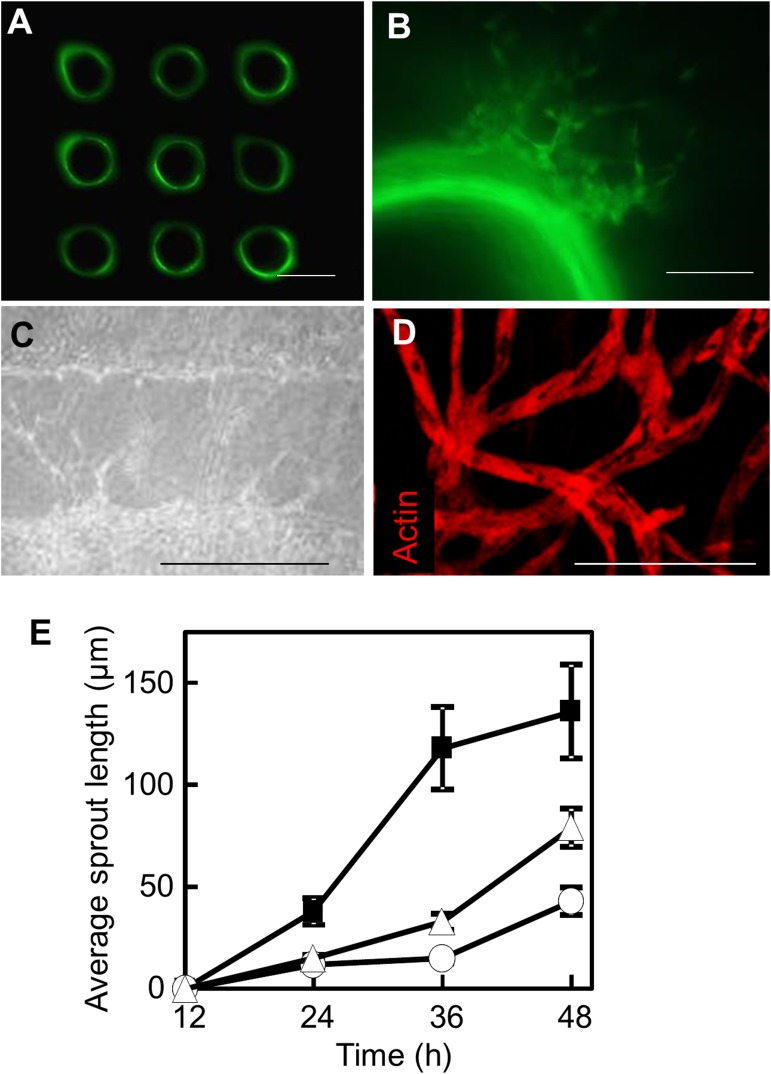
Synergistic effects of PMA and perfusion on endothelial cell sprouting. (A) Cross-sections of 3D-aligned HUVEC-enveloped microchannels. GFP-HUVECs were transferred to the internal surfaces by using the multi-needle. (B) HUVEC sprouting after 48 h of culture under perfusion at 10 μL/min and 50 ng/mL PMA. (C) Sprouts extended toward neighboring microchannels and bridged them after as few as 7 d. (D) Confocal microscopy image of luminal structures. (E) Quantification of the sprout length. Static culture in the absence of PMA (○), perfusion culture in the absence of PMA (Δ), and perfusion culture in the presence of PMA (■). Error bars indicate standard deviations calculated from three independent experiments. Scale bars indicate 500 μm in (A, C), and 100 μm in (B, D).

HUVEC sprouting in the presence of perfusion at 10 μL/min and 50 ng/mL PMA was observed at 6 h of culture, and sprout length reached 100 μm after 48 h. Quantitative analysis revealed that the average sprout length after 48 h of culture increased by up to 84% in response to the addition of PMA and by up to 216% when cells were also subjected to shear stress (Fig 5E). At 7 d of perfusion culture, the sprouting areas from the neighboring microchannels crossed over each other (Fig 5C), and tubular structures were observed, as seen in typical angiogenesis assays in which collagen sandwiches were used (Fig 5D).

This rapid approach for endothelialized microchannel fabrication may be beneficial, especially when proliferative parenchymal cells such as iPS-derived hepatic lineage cells are encapsulated in the collagen gel to engineer vascularized 3D cell-dense tissues. We expect that tissues and organs with greater complexity could be fabricated through self-organization of endothelial cells and parenchymal cells under perfusion culture conditions (S1 Fig).

## Conclusions

A simple and rapid approach to fabricate vascular-like structures and accelerate their sprouting in hydrogel was presented. Given that parenchymal cells will be encapsulated in hydrogel when engineering 3D cell-dense tissues, rapid processes to engineer perfusable vasculatures and induce luminal networks are of fundamental importance. By electrochemically transferring endothelial cells from cylindrical needles, endothelialized microchannels were fabricated and culture medium perfusion began within 30 min. Although endothelial cells have a propensity to form luminal structures in hydrogel, the process was relatively slow, taking >14 d to reach the regions where neighboring vascular-like structures aligned at 500-μm intervals were connected. Shear stress and PMA stimuli were effective in acceleration of sprouting. These stimuli synergistically accelerated sprouting and the average length of sprouts at 2 d of culture was more than twice that in the absence of stimuli. At 7 d of culture, the sprouting regions from neighboring microchannels began to cross over each other. Taken together, these results suggest that this is an effective approach to fabricate vasculatures to reconstruct solid organs such as the liver *in vitro*.

## Supporting Information

S1 Fig Approach for engineering vascularized 3D liver tissues. The strategy involves encapsulation of proliferative cells such as iPS-derived hepatic cells in a collagen gel. Spontaneous organization of proliferative cells and sprouting endothelial cells is expected during perfusion culture, potentially leading to vascularized 3D tissue development.
(TIF)

S1 Movie Visualization of vascular networks. Luminal structures of HUVECs (green) transferred to internal surfaces of microchannels in collagen gel. Luminal structures and branching points are represented by white tracing lines and blue spheres, respectively.
(MP4)

## References

[1] Langer R, Vacanti JP. TISSUE ENGINEERING. Science. 1993;260(5110):920–6. 10.1126/science.8493529 . 8493529

[2] Koh CJ, Atala A. Tissue engineering, stem cells, and cloning: Opportunities for regenerative medicine. J Am Soc Nephrol. 2004;15(5):1113–25. 10.1097/01.asn.0000119683.59068.f0 . 15100351

[3] Okano T, Matsuda T. Muscular tissue engineering: capillary-incorporated hybrid muscular tissues in vivo tissue culture. Cell Transplantation. 1998;7(5):435–42. 10.1016/S0963-6897(98)00030-X 9786063

[4] Brittberg M, Lindahl A, Nilsson A, Ohlsson C, Isaksson O, Peterson L. Treatment of Deep Cartilage Defects in the Knee with Autologous Chondrocyte Transplantation. New England Journal of Medicine. 1994;331(14):889–95. 10.1056/NEJM199410063311401 . 8078550

[5] Nishida K, Yamato M, Hayashida Y, Watanabe K, Yamamoto K, Adachi E, et al Corneal Reconstruction with Tissue-Engineered Cell Sheets Composed of Autologous Oral Mucosal Epithelium. New England Journal of Medicine. 2004;351(12):1187–96. 10.1056/NEJMoa040455 15371576

[6] Frerich B, Lindemann N, Kurtz-Hoffmann J, Oertel K. In vitro model of a vascular stroma for the engineering of vascularized tissues. International Journal of Oral and Maxillofacial Surgery. 2001;30(5):414–20. 10.1054/ijom.2001.0130 11720044

[7] Chen X, Aledia AS, Ghajar CM, Griffith CK, Putnam AJ, Hughes CC, et al Prevascularization of a fibrin-based tissue construct accelerates the formation of functional anastomosis with host vasculature. Tissue Eng Part A. 2009;15(6):1363–71. Epub 2008/11/04. 10.1089/ten.tea.2008.0314 18976155 PMC2792096

[8] Koike N, Fukumura D, Gralla O, Au P, Schechner JS, Jain RK. Tissue engineering: Creation of long-lasting blood vessels. Nature. 2004;428(6979):138–9. 15014486 10.1038/428138a

[9] Nakatsu MN, Sainson RCA, Aoto JN, Taylor KL, Aitkenhead M, Pérez-del-Pulgar S, et al Angiogenic sprouting and capillary lumen formation modeled by human umbilical vein endothelial cells (HUVEC) in fibrin gels: the role of fibroblasts and Angiopoietin-1. Microvascular Research. 2003;66(2):102–12. 10.1016/s0026-2862(03)00045-1 12935768

[10] Bouloumie A, Drexler HCA, Lafontan M, Busse R. Leptin, the Product of Ob Gene, Promotes Angiogenesis. Circulation Research. 1998;83(10):1059–66. 10.1161/01.res.83.10.1059 9815153

[11] Ling Y, Rubin J, Deng Y, Huang C, Demirci U, Karp JM, et al A cell-laden microfluidic hydrogel. Lab on a Chip. 2007;7(6):756–62. 10.1039/B615486g . 17538718

[12] Golden AP, Tien J. Fabrication of microfluidic hydrogels using molded gelatin as a sacrificial element. Lab Chip. 2007;7(6):720–5. Epub 2007/06/01. 10.1039/b618409j . 17538713

[13] Cuchiara MP, Allen ACB, Chen TM, Miller JS, West JL. Multilayer microfluidic PEGDA hydrogels. Biomaterials. 2010;31(21):5491–7. 10.1016/j.biomaterials.2010.03.031 20447685

[14] Miller JS, Stevens KR, Yang MT, Baker BM, Nguyen DH, Cohen DM, et al Rapid casting of patterned vascular networks for perfusable engineered three-dimensional tissues. Nat Mater. 2012;11(9):768–74. Epub 2012/07/04. doi: nmat3357 [pii] doi: 10.1038/nmat3357. 22751181 22751181 10.1038/nmat3357 PMC3586565

[15] Therriault D, White SR, Lewis JA. Chaotic mixing in three-dimensional microvascular networks fabricated by direct-write assembly. Nat Mater. 2003;2(4):265–71. 12690401 10.1038/nmat863

[16] Bellan LM, Singh SP, Henderson PW, Porri TJ, Craighead HG, Spector JA. Fabrication of an artificial 3-dimensional vascular network using sacrificial sugar structures. Soft Matter. 2009;5(7):1354–7.

[17] Takei T, Sakai S, Ono T, Ijima H, Kawakami K. Fabrication of endothelialized tube in collagen gel as starting point for self-developing capillary-like network to construct three-dimensional organs in vitro. Biotechnology and Bioengineering. 2006;95(1):1–7. 10.1002/Bit.20903 . 16604522

[18] Martou G, O’Blenes CA, Huang N, McAllister SE, Neligan PC, Ashrafpour H, et al. Development of an in vitro model for study of the efficacy of ischemic preconditioning in human skeletal muscle against ischemia-reperfusion injury2006 2006-11-01 00:00:00. 1335–42 p. 10.1152/japplphysiol.00278.2006 17043328

[19] Bhogal RH, Curbishley SM, Weston CJ, Adams DH, Afford SC. Reactive oxygen species mediate human hepatocyte injury during hypoxia/reoxygenation. Liver Transplantation. 2010;16(11):1303–13. 10.1002/lt.22157 21031546

[20] Inaba R, Khademhosseini A, Suzuki H, Fukuda J. Electrochemical desorption of self-assembled monolayers for engineering cellular tissues. Biomaterials. 2009;30(21):3573–9. 10.1016/j.biomaterials.2009.03.045 . 19362363

[21] Seto Y, Inaba R, Okuyama T, Sassa F, Suzuki H, Fukuda J. Engineering of capillary-like structures in tissue constructs by electrochemical detachment of cells. Biomaterials. 2010;31(8):2209–15. 10.1016/j.biomaterials.2009.11.104 . 20022631

[22] Mochizuki N, Kakegawa T, Osaki T, Sadr N, Kachouie NN, Suzuki H, et al Tissue engineering based on electrochemical desorption of an RGD-containing oligopeptide. Journal of tissue engineering and regenerative medicine. 2011;7(3):236–43. Epub 2011/12/14. 10.1002/term.519 . 22162306

[23] Kakegawa T, Mochizuki N, Sadr N, Suzuki H, Fukuda J. Cell-adhesive and cell-repulsive zwitterionic oligopeptides for micropatterning and rapid electrochemical detachment of cells. Tissue Eng Part A. 2013;19(1–2):290–8. Epub 2012/08/03. 10.1089/ten.TEA.2011.0739 22853640 PMC3530950

[24] Millauer B, Wizigmann-Voos S, Schnürch H, Martinez R, Møller NPH, Risau W, et al High affinity VEGF binding and developmental expression suggest Flk-1 as a major regulator of vasculogenesis and angiogenesis. Cell. 1993;72(6):835–46. 10.1016/0092-8674(93)90573-9 7681362

[25] Folkman J. Angiogenesis In: Jaffe E, editor. Biology of Endothelial Cells. Developments in Cardiovascular Medicine. 27: Springer US; 1984 p. 412–28.

[26] Powers CJ, McLeskey SW, Wellstein A. Fibroblast growth factors, their receptors and signaling. Endocr-Relat Cancer. 2000;7(3):165–97. 10.1677/erc.0.0070165 . 11021964

[27] Gerhardt H, Betsholtz C. Endothelial-pericyte interactions in angiogenesis. Cell Tissue Res. 2003;314(1):15–23. 10.1007/s00441-003-0745-x . 12883993

[28] Gerhardt H, Golding M, Fruttiger M, Ruhrberg C, Lundkvist A, Abramsson A, et al VEGF guides angiogenic sprouting utilizing endothelial tip cell filopodia. The Journal of Cell Biology. 2003;161(6):1163–77. 10.1083/jcb.200302047 12810700 PMC2172999

[29] Hellstrom M, Phng LK, Hofmann JJ, Wallgard E, Coultas L, Lindblom P, et al Dll4 signalling through Notch1 regulates formation of tip cells during angiogenesis. Nature. 2007;445(7129):776–80. 10.1038/Nature05571 . 17259973

[30] Strair R. Study of 12-O-Tetradecanoylphorbol-13-acetate in Treating Patients With Hematologic Cancer or Bone Marrow Disorder. ClinicalTrials.gov Identifier:NCT00004058, University of Medicine and Dentistry of New Jersey. Available at http://clinicaltrials.gov/ct2/show/NCT00004058. Accessed January 2010. 1999.

[31] Duda DG, Fukumura D, Jain RK. Role of eNOS in neovascularization: NO for endothelial progenitor cells. Trends Mol Med. 2004;10(4):143–5. 10.1016/j.molmed.2004.02.001 . 15162796

[32] Song JW, Munn LL. Fluid forces control endothelial sprouting. Proceedings of the National Academy of Sciences of the United States of America. 2011;108(37):15342–7. 10.1073/pnas.1105316108 21876168 PMC3174629

[33] Chen SF, Cao ZQ, Jiang SY. Ultra-low fouling peptide surfaces derived from natural amino acids. Biomaterials. 2009;30(29):5892–6. 10.1016/j.biomaterials.2009.07.001 . 19631374

[34] Huang X, Zauscher S, Klitzman B, Truskey GA, Reichert WM, Kenan DJ, et al Peptide Interfacial Biomaterials Improve Endothelial Cell Adhesion and Spreading on Synthetic Polyglycolic Acid Materials. Annals of Biomedical Engineering. 2010;38(6):1965–76. 10.1007/s10439-010-9986-5 . 20300848

[35] Bayless KJ, Kwak HI, Su SC. Investigating endothelial invasion and sprouting behavior in three-dimensional collagen matrices. Nat Protoc. 2009;4(12):1888–98. Epub 2009/12/17. doi: nprot.2009.221 [pii] 10.1038/nprot.2009.221 . 20010936

[36] Morimoto Y, Kato-Negishi M, Onoe H, Takeuchi S. Three-dimensional neuron–muscle constructs with neuromuscular junctions. Biomaterials. 2013;34(37):9413–9. 10.1016/j.biomaterials.2013.08.062 24041425

[37] Ayres C, Bowlin GL, Henderson SC, Taylor L, Shultz J, Alexander J, et al Modulation of anisotropy in electrospun tissue-engineering scaffolds: Analysis of fiber alignment by the fast Fourier transform. Biomaterials. 2006;27(32):5524–34. 10.1016/j.biomaterials.2006.06.014 16859744 PMC2929953

[38] Seano G, Chiaverina G, Gagliardi PA, di Blasio L, Sessa R, Bussolino F, et al Modeling human tumor angiogenesis in a three-dimensional culture system. Blood. 2013;121(21):e129–37. 10.1182/blood-2012-08-452292 . 23471306

[39] Hecker E. Phorbol esters from croton oil chemical nature and biological activities. Naturwissenschaften. 1967;54(11):282–4. 10.1007/BF00620887 5589922

[40] Gross JL, Moscatelli D, Rifkin DB. Increased capillary endothelial cell protease activity in response to angiogenic stimuli in vitro. Proc Natl Acad Sci U S A. 1983;80(9):2623–7. Epub 1983/05/01. 6302697 10.1073/pnas.80.9.2623 PMC393879

[41] Montesano R, Orci L. Tumor-promoting phorbol esters induce angiogenesis in vitro. Cell. 1985;42(2):469–77. doi: 10.1016/0092-8674(85)90104-7. 2411423 2411423 10.1016/0092-8674(85)90104-7

[42] Park IC, Park MJ, Rhee CH, Lee JI, Choe TB, Jang JJ, et al Protein kinase C activation by PMA rapidly induces apoptosis through caspase-3/CPP32 and serine protease(s) in a gastric cancer cell line. Int J Oncol. 2001;18(5):1077–83. . 11295059 10.3892/ijo.18.5.1077

[43] Busuttil V, Bottero V, Frelin C, Imbert V, Ricci JE, Auberger P, et al Blocking NF-kappaB activation in Jurkat leukemic T cells converts the survival agent and tumor promoter PMA into an apoptotic effector. Oncogene. 2002;21(20):3213–24. 12082637 10.1038/sj.onc.1205433

[44] Karsan A, Yee E, Poirier GG, Zhou P, Craig R, Harlan JM. Fibroblast growth factor-2 inhibits endothelial cell apoptosis by Bcl-2-dependent and independent mechanisms. The American journal of pathology. 1997;151(6):1775–84. 9403728 PMC1858363

[45] Pries AR, Secomb TW, Gaehtgens P. Design principles of vascular beds. Circ Res. 1995;77(5):1017–23. . 7554136 10.1161/01.res.77.5.1017

[46] Langille LB. Remodeling of Developing and Mature Arteries: Endothelium, Smooth Muscle, and Matrix. Journal of Cardiovascular Pharmacology. 1993;21:S11–S7. . 7681126 10.1097/00005344-199321001-00003

[47] Yamamoto K, Takahashi T, Asahara T, Ohura N, Sokabe T, Kamiya A, et al. Proliferation, differentiation, and tube formation by endothelial progenitor cells in response to shear stress2003 2003-11-01 00:00:00. 2081–8 p. 10.1152/japplphysiol.00232.2003 12857765

[48] Tkachenko E, Gutierrez E, Ginsberg MH, Groisman A. An easy to assemble microfluidic perfusion device with a magnetic clamp. Lab on a Chip. 2009;9(8):1085–95. 10.1039/B812184B 19350090 PMC2742503

[49] Risau W. Mechanisms of angiogenesis. Nature. 1997;386(6626):671–4. 10.1038/386671a0 . 9109485

[50] Baum O, Da Silva-Azevedo L, Willerding G, Wockel A, Planitzer G, Gossrau R, et al Endothelial NOS is main mediator for shear stress-dependent angiogenesis in skeletal muscle after prazosin administration. Am J Physiol Heart Circ Physiol. 2004;287(5):H2300–8. Epub 2004/07/03. 10.1152/ajpheart.00065.200400065.2004 . 15231496

[51] Fulton D, Gratton JP, McCabe TJ, Fontana J, Fujio Y, Walsh K, et al Regulation of endothelium-derived nitric oxide production by the protein kinase Akt. Nature. 1999;399(6736):597–601. 10.1038/21218 10376602 PMC3637917

[52] Zhang W, Liu HT. MAPK signal pathways in the regulation of cell proliferation in mammalian cells. Cell research. 2002;12(1):9–18. 10.1038/sj.cr.7290105 . 11942415

